# On the Evolutionary History, Population Genetics and Diversity among Isolates of *Salmonella* Enteritidis PFGE Pattern JEGX01.0004

**DOI:** 10.1371/journal.pone.0055254

**Published:** 2013-01-30

**Authors:** Marc W. Allard, Yan Luo, Errol Strain, James Pettengill, Ruth Timme, Charles Wang, Cong Li, Christine E. Keys, Jie Zheng, Robert Stones, Mark R. Wilson, Steven M. Musser, Eric W. Brown

**Affiliations:** 1 Office of Regulatory Science, Center for Food Safety and Applied Nutrition, Food and Drug Administration, College Park, Maryland, United States of America; 2 Office of Food Defense, Communications, and Emergency Response, Center for Food Safety and Applied Nutrition, Food and Drug Administration, College Park, Maryland, United States of America; 3 Food and Environment Research Agency, Sand Hutton, York, United Kingdom; 4 Forensic Science Program, Western Carolina University, Cullowhee, North Carolina, United States of America; Facultad de Medicina, Uruguay

## Abstract

Facile laboratory tools are needed to augment identification in contamination events to trace the contamination back to the source (traceback) of *Salmonella enterica* subsp. *enterica* serovar Enteritidis (*S.* Enteritidis). Understanding the evolution and diversity within and among outbreak strains is the first step towards this goal. To this end, we collected 106 new S. Enteriditis isolates within S. Enteriditis Pulsed-Field Gel Electrophoresis (PFGE) pattern JEGX01.0004 and close relatives, and determined their genome sequences. Sources for these isolates spanned food, clinical and environmental farm sources collected during the 2010 *S.* Enteritidis shell egg outbreak in the United States along with closely related serovars, *S.* Dublin, *S.* Gallinarum biovar Pullorum and *S.* Gallinarum. Despite the highly homogeneous structure of this population, *S.* Enteritidis isolates examined in this study revealed thousands of SNP differences and numerous variable genes (n = 366). Twenty-one of these genes from the lineages leading to outbreak-associated samples had nonsynonymous (causing amino acid changes) changes and five genes are putatively involved in known *Salmonella* virulence pathways. While chromosome synteny and genome organization appeared to be stable among these isolates, genome size differences were observed due to variation in the presence or absence of several phages and plasmids, including phage RE-2010, phage P125109, plasmid pSEEE3072_19 (similar to pSENV), plasmid pOU1114 and two newly observed mobile plasmid elements pSEEE1729_15 and pSEEE0956_35. These differences produced modifications to the assembled bases for these draft genomes in the size range of approximately 4.6 to 4.8 mbp, with *S.* Dublin being larger (∼4.9 mbp) and *S.* Gallinarum smaller (4.55 mbp) when compared to *S.* Enteritidis. Finally, we identified variable *S.* Enteritidis genes associated with virulence pathways that may be useful markers for the development of rapid surveillance and typing methods, potentially aiding in traceback efforts during future outbreaks involving *S.* Enteritidis PFGE pattern JEGX01.0004.

## Introduction

The accurate subtyping and subsequent clustering of bacterial isolates associated with a foodborne outbreak event is important for successful investigation and eventual traceback to a specific food or environmental source. However, clonally derived strains, common within *Salmonella enterica* subsp. *enterica* serovar Enteritidis (*S.* Enteritidis), confound epidemiological investigations because of the limited genetic differentiation of these strains [1]–[9]. Existing approaches often lack the resolution for separating tightly linked bacterial isolates such as those originating from *S.* Enteritidis. In response to such events, federal public health, academic and industry food safety laboratories are exploring next-generation sequencing (NGS) technologies to investigate complex and challenging outbreak scenarios [10]–[18]. Recent examples in the literature illustrate the ability of NGS to detect variation within otherwise indistinguishable isolates [19]–[23]. These efforts have identified micro-evolutionary differences that genetically link clinical isolates, outbreak isolates found in foods, and their environmental counterparts in *Salmonella* [19]–[24], *Escherichia coli* [25]–[27], *Vibrio* [28]–[30] as well as numerous other bacteria [31]–[37]. Our genomics laboratory and others have successfully applied these NGS approaches to a case study of *S.* Montevideo in spiced Italian-style meats [19]–[21] where it was determined that the methods and results were reproducible. Moreover, extensive data mining within these novel genomes should yield novel genetic targets to augment investigations during outbreaks of highly clonal *Salmonella* pathogens.

*S.* Enteritidis remains a significant pathogen and a substantial threat to the food supply. It also represents one of the most genetically homogeneous serotypes of *Salmonella*, and certain clonal lineages remain intractable to differentiation by commonly used conventional subtyping methods [38]–[46]. The unusual genetic homogeneity observed among certain lineages of *S.* Enteritidis strains remains intriguing. Recent population genetic studies suggest that most *S.* Enteritidis strains belong to a single multilocus genotype [4]–[6]. A subpopulation of this clone was shown to associate more frequently with egg-related salmonellosis and clinical illness [4]. Thus, specific requirements for colonization and survival in infected poultry may select for only a few genotypes of *S.* Enteritidis in the poultry environment. The random amplification of polymorphic DNA (RAPD), real-time polymerase chain reaction (RT-PCR), and Phage typing (PT) methods [2], [7], [9], [45], [46] from diverse isolates within *S.* Enteritidis have revealed only a limited amount of genetic variation. More recently, more resolved discriminations of these salmonellae have been reported using rapidly-evolving CRISPR elements [5], [17]. Conversely, rather than targeting a subset or region of variation in the S. Enteritidis chromosome, whole genome sequencing (WGS) will capture all of the genetic variation that exists among these highly clonal lineages. To date, only a few strains of *S.* Enteritidis are available as complete genomes [47]–[48] along with close relatives *S.* Gallinarum [11] and *S.* Gallinarum biovar Pullorum [49]. These isolates have genome sizes around 4.7 mbp. The basic pan genomes are described in these initial studies, but currently, there are no published NCBI draft comparative genomes or associated manuscripts describing variation within *S.* Enteritidis. In this study, we describe the natural genetic variation within *S.* Enteritidis isolates associated with a widespread egg contamination event and retaining pulsed-field gel electrophoresis (PFGE) pattern JEGX01.0004 and analyze the comparative evolutionary genetics within this important foodborne pathogen and several of its closest relatives.

In 2010, the Centers for Disease Control and Prevention (CDC) along with many state laboratories identified a nationwide increase in *S.* Enteritidis isolates submitted to PulseNet (http://www.cdc.gov/salmonella/enteritidis/). Epidemiological investigations suggested that shell eggs were the most likely source of this increase. FDA, CDC, and state partners conducted traceback investigations and found many of the restaurants involved received shell eggs from a single company (http://www.fda.gov/food/newsevents/whatsnewinfood/ucm222684.htm). As a result, on August 13, 2010, one egg producer initiated a nationwide voluntary recall of shell eggs that had been sold to distributors and wholesalers in 22 states and Mexico. A record 380 million shell eggs were recalled under many different brand names. On August 19, a second egg producer initiated an additional recall of eggs that went to grocery stores, distributors, and wholesalers in 14 states. The second producer shared a contaminated feed supply with the first and was geographically nearby. In all, more than 500 million eggs were involved during this nationwide recall.

The primary goal of this study was to examine the genetic variability of isolates collected during the 2010 *S.* Enteritidis shell egg outbreak within the PFGE pattern JEGX01.0004, a pattern comprising over 40% of all of the *S.* Enteritidis isolates submitted to the national database. We also included several other isolates with similar PFGE patterns to JEGX01.0004 found in the associated egg-farm environment. We went on to describe the genetic diversity and evolutionary history of 106 new draft genomes for this virulent pathogen within this narrow but important sampling of *S.* Enteritidis diversity. As a result, we were able to provide new genetic targets useful for distinguishing *S.* Enteritidis isolates otherwise indistinguishable by several current methodologies. Once validated, these new SNP targets can be interrogated using widely available DNA sequencing through capillary electrophoresis (CE), short-read pyrosequencing, real-time PCR, or mass spectrometry of PCR amplicons. Finally, this study evaluates the potential use of targeted genomic sequencing with next generation sequencing (NGS) for rapidly resolving future *S.* Enteritidis outbreaks in eggs.

## Materials and Methods

### Salmonella Enteritidis strains

A set of 67 food, environmental, and clinical *S.* Enteritidis isolates collected from farms and egg sources linked to the 2010 egg contamination event was included for whole genome sequencing. Specifically, 36 *S.* Enteritidis isolates, originating from environmental swabs, were collected directly from various farm sources implicated in the contamination event (e.g., egg wash water). Four *S.* Enteritidis were isolated directly from shell eggs, liquid eggs, or other egg-containing food sources known to be contaminated during this time period. Two *S.* Enteritidis isolates were obtained directly from chicken feed or components thereof at the implicated farms. An additional 25 clinical isolates, collected during the time of the egg contamination event (2010) and retaining common PFGE patterns to the egg *S.* Enteritidis isolates, were kindly provided by the Centers for Disease Control and included for sequencing. In addition, 39 isolates, collected earlier in time and unrelated to the contamination event, were added as reference *S.* Enteritidis for the WGS analysis. These included 13 isolates with two-enzyme matching PFGE patterns, seven single-enzyme matching patterns, indistinguishable in either the primary (XbaI n = 3) or secondary (BlnI n = 4) enzyme, and 19 isolates with no common PFGE patterns to the contamination event. These isolates also were used to further investigate the phylogenetic utility of phage-typing. Included in this group of 39 were 10 of unknown PT and, 14 of historical PT8 isolates. The remainder were 15 isolates of ***S.*** Enteritidis from ten other diverged PTs such as PT1, 21, 2, 4, 14b, 13, 13a, 23, 28 and 35. ***S.*** Enteritidis strains were phage-typed by previously described methods [2] at the National Microbiology Laboratory, Canadian Science Centre for Human and Animal Health, Winnipeg, Manitoba, Canada. Strains that reacted with phages but retained unrecognizable lytic patterns were atypical and were designated atypical or RDNC (reacts but does not conform). Specific PFGE pattern names, PTs, and other metadata associated with the *S.* Enteritidis strains are listed in Table 1 (PTs are included in the tree label names).

**Table 1:** Metadata associated with the isolates examined in this study.

Isolates included in this study						Accession no(s)		presense of mobile elements						
Tree Labels	Salmonella enterica subsp. enterica serovar and strain	Collection location	Isolation source	Collection date	PFGE Pattern (Primary enzyme_secondary enzyme)	BioProject	WGS	Reference	RE-2010 Phage	P125109 Phage	plasmid pOU1114	plasmid pSEEE1729_15	plasmid pSEEE0956_35	plasmid pSEEE3072_19
Gallinarum-SEEG9184	Galinarum str. 9184	-	-	-	NA_NA	41263	AHUH00000000	current						
Dublin-SEEDSL	Dublin str. HWS51	-	-	-	NA_NA	41463	AHUJ00000000	current						
Dublin-SEEDHWS	Dublin str. SL1438	-	-	-	NA_NA	41465	AHUK00000000	current						
Ent.PT8-GA-SEE8A	Enteritidis str. SE8a	USA:GA	-	-	NA_NA	41915	AHUL00000000	current	**+**				**+**	
Ent.PT14b-TN-chicken breast-SE20037	Enteritidis str. 20037	USA:TN	Chicken Breast	-	NA_NA	41917	AHUM00000000	current	**+**				**+**	
Ent.PT8-ME-chicken ovary-SEE10	Enteritidis str. SE10	USA:ME	Chicken Ovary	-	JEGX01.0004_JEGA26.0002	41919	AHUN00000000	current	**+**				**+**	
Ent.PT4-Scotland-chicken liver-SEE436	Enteritidis str. 436	Scotland	Chicken Liver	-	JEGX01.0002_NA	41921	AHUO00000000	current		+			**+**	
Ent.PT14b-Meixco-poultry-<1?show=[sr]?>SEE18569	Enteritidis str. 18569	Meixco	Poultry	-	JEGX01.0002_NA	41929	AHUP00000000	current		+			**+**	
Ent.PT13-GA-chicken-SEE13	Enteritidis str. 13-1	USA:GA	Chicken	-	JEGX01.0005_NA	41931	AHUQ00000000	current	**+**				**+**	
Ent.PT23-GA-SEE23	Enteritidis str. PT23	USA:GA	-	-	JEGX01.0005_NA	41933	AHUR00000000	current	**+**				**+**	
Ent.PT28-MD-ground turkey-SEE22704	Enteritidis str. 22704	USA:MD	Ground Turkey	-	NA_NA	42901	ALFD00000000	current	**+**				**+**	
Ent.PT21-MD-ground turkey-SEE30663	Enteritidis str. SE30663	USA:MD	Ground Turkey	-	JEGX01.0019_JEGA26.0010	42905	ALFE00000000	current		+			**+**	
Ent.PT1-China-chicken-SEECHS44	Enteritidis str. CHS44	China	Chicken	-	NA_NA	42907	ALFF00000000	current		+				
Ent-CO-2010-clinical-SEEE1882	Enteritidis str. CDC 2010K-1882	USA:CO	Clinical	2010-07-15	JEGX01.0004_JEGA26.0002	51991	ALFG00000000	current	**+**			**+**	**+**	
Ent-CO-2010-rattle snake cake-SEEE1884	Enteritidis str. CDC 2010K-1884	USA:CO	Rattlesnake Cake	2010-08-02	JEGX01.0004_JEGA26.0002	51993	ALFH00000000	current	**+**			**+**	**+**	
Ent-CA-2010-clinical-SEEE1594	Enteritidis str. CDC 2010K-1594	USA:CA	Clinical	2010-05-23	JEGX01.0004_JEGA26.0002	51995	ALFI00000000	current	**+**			**+**	**+**	
Ent-MN-2010-clinical-SEEE1566	Enteritidis str. CDC 2010K-1566	USA:MN	Clinical	2010-05-27	JEGX01.0004_JEGA26.0002	51997	ALFJ00000000	current	**+**				**+**	
Ent-MN-2010-clinical-SEEE1580	Enteritidis str. CDC 2010K-1580	USA:MN	Clinical	2010-07-10	JEGX01.0004_JEGA26.0002	51999	ALFK00000000	current	**+**				**+**	
Ent-CA-2010-clinical-SEEE1543	Enteritidis str. CDC 2010K-1543	USA:CA	Clinical	2010-05-23	JEGX01.0004_JEGA26.0002	52001	ALFL00000000	current	**+**				**+**	
Ent-CA-2010-clinical-SEEE1441	Enteritidis str. CDC 2010K-1441	USA:CA	Clinical	2010-06-03	JEGX01.0004_JEGA26.0002	52003	ALFM00000000	current	**+**				**+**	
Ent-TX-2010-clinical-SEEE1810	Enteritidis str. CDC 2010K-1810	USA:TX	Clinical	2010-06-02	JEGX01.0004_JEGA26.0002	52005	ALFN00000000	current	**+**			**+**	**+**	
Ent-IA-2010-clinical-SEEE1558	Enteritidis str. CDC 2010K-1558	USA:IA	Clinical	2010-07-04	JEGX01.0004_JEGA26.0002	52371	ALFO00000000	current	**+**			**+**	**+**	
Ent-NC-2010-clinical-SEEE1018	Enteritidis str. CDC 2010K-1018	USA:NC	Clinical	2010-04-27	JEGX01.0004_JEGA26.0002	52373	ALFP00000000	current	**+**				**+**	
Ent-NC-2010-meringue-SEEE1010	Enteritidis str. CDC 2010K-1010	USA:NC	Meringue	2010-04-28	JEGX01.0108_JEGA26.0002	52375	ALFQ00000000	current	**+**				**+**	
Ent-NV-2010-clinical-SEEE1729	Enteritidis str. CDC 2010K-1729	USA:NV	Clinical	2010-07-24	JEGX01.0004_JEGA26.0002	52377	ALFR00000000	current	**+**			**+**	**+**	
Ent-OH-2010-clinical-SEEE0895	Enteritidis str. CDC 2010K-0895	USA:OH	Clinical	2010-05-02	JEGX01.0004_JEGA26.0002	52379	ALFS00000000	current	**+**			**+**	**+**	
Ent-OH-2010-mexican meal-SEEE0899	Enteritidis str. CDC 2010K-0899	USA:OH	Mexican Meal	2010-05-12	JEGX01.0004_JEGA26.0002	52381	ALFT00000000	current	**+**			**+**	**+**	
Ent-PA-2010-clinical-SEEE1457	Enteritidis str. CDC 2010K-1457	USA:PA	Clinical	2010-05-27	JEGX01.0004_JEGA26.0002	52383	ALFU00000000	current	**+**				**+**	
Ent-WI-2010-clinical-SEEE1747	Enteritidis str. CDC 2010K-1747	USA:WI	Clinical	2010-06-26	JEGX01.0004_JEGA26.0002	52385	ALFV00000000	current	**+**			**+**	**+**	
Ent-OH-2010-clinical-SEEE0968	Enteritidis str. CDC 2010K-0968	USA:OH	Clinical	2010-05-12	JEGX01.0004_JEGA26.0002	52491	ALFW00000000	current	**+**			**+**	**+**	
Ent-CA-2010-clinical-SEEE1444	Enteritidis str. CDC 2010K-1444	USA:CA	Clinical	2010-06-12	JEGX01.0004_JEGA26.0002	52495	ALFX00000000	current	**+**			**+**	**+**	
Ent-CA-2010-clinical-SEEE1445	Enteritidis str. CDC 2010K-1445	USA:CA	Clinical	2010-06-09	JEGX01.0004_JEGA26.0002	52497	ALFY00000000	current	**+**				**+**	
Ent-IA-2010-clinical-SEEE1559	Enteritidis str. CDC 2010K-1559	USA:IA	Clinical	2010-07-03	JEGX01.0004_JEGA26.0002	52499	ALFZ00000000	current	**+**			**+**	**+**	
Ent-MN-2010-clinical-SEEE1565	Enteritidis str. CDC 2010K-1565	USA:MN	Clinical	2010-05-30	JEGX01.0004_JEGA26.0002	52501	ALGA00000000	current	**+**				**+**	
Ent-TX-2010-clinical-SEEE1808	Enteritidis str. CDC 2010K-1808	USA:TX	Clinical	2010-06-01	JEGX01.0004_JEGA26.0002	52503	ALGB00000000	current	**+**			**+**	**+**	
Ent-TX-2010-clinical-SEEE1811	Enteritidis str. CDC 2010K-1811	USA:TX	Clinical	2010-06-04	JEGX01.0004_JEGA26.0002	52505	ALGC00000000	current	**+**			**+**	**+**	
Ent-OH-2010-clinical-SEEE0956	Enteritidis str. CDC 2010K-0956	USA:OH	Clinical	2010-05-04	JEGX01.0004_JEGA26.0002	52507	ALGD00000000	current	**+**			**+**	**+**	
Ent-PA-2010-clinical-SEEE1455	Enteritidis str. CDC 2010K-1455	USA:PA	Clinical	2010-05-26	JEGX01.0004_JEGA26.0002	52509	ALGE00000000	current	**+**				**+**	
Ent-MN-2010-clinical-SEEE1575	Enteritidis str. CDC 2010K-1575	USA:MN	Clinical	2010-07-03	JEGX01.0004_JEGA26.0002	52511	ALGF00000000	current	**+**				**+**	
Ent-NV-2010-clinical-SEEE1725	Enteritidis str. CDC 2010K-1725	USA:NV	Clinical	2010-07-13	JEGX01.0004_JEGA26.0002	52513	ALGG00000000	current	**+**			**+**	**+**	
Ent-WI-2010-clinical-SEEE1745	Enteritidis str. CDC 2010K-1745	USA:WI	Clinical	2010-06-25	JEGX01.0004_JEGA26.0002	52515	ALGH00000000	current	**+**			**+**	**+**	
Ent-TN-2010-clinical-SEEE1791	Enteritidis str. CDC 2010K-1791	USA:TN	Clinical	2010-06-13	JEGX01.0004_JEGA26.0002	52517	ALGI00000000	current	**+**				**+**	
Ent-TN-2010-clinical-SEEE1795	Enteritidis str. CDC 2010K-1795	USA:TN	Clinical	2010-06-15	JEGX01.0004_JEGA26.0002	52519	ALGJ00000000	current	**+**				**+**	
Ent-IA-2010-bulk bone meal-SEEE6709	Enteritidis str. 576709	USA:IA	Bulk Bone Meal	2010-08-14	JEGX01.0004_JEGA26.0002	52613	ALGK00000000	current	**+**			**+**	**+**	
Ent-IA-2010-env swab-SEEE3139	Enteritidis str. 622731-39	USA:IA	Environmental Swab	2010-08-13	JEGX01.0004_JEGA26.0002	52615	ALEI00000000	(Timme, 2012)	**+**			**+**	**+**	
Ent-IA-2010-egg wash water-SEEE0166	Enteritidis str. 639016-6	USA:IA	Egg Wash Water	2010-08-19	JEGX01.0004_JEGA26.0002	52617	ALEJ00000000	(Timme, 2012)	**+**			**+**	**+**	
Ent-IA-2010-chicken feed-SEEE0631	Enteritidis str. 640631	USA:IA	Chicken Feed-Developer Pullet	2010-08-17	JEGX01.0004_JEGA26.0002	52619	ALEK00000000	(Timme, 2012)	**+**				**+**	
Ent-IA-2010-env swab-SEEE9058	Enteritidis str. 635290-58	USA:IA	Environmental Swab	2010-08-16	JEGX01.0004_JEGA26.0002	52621	ALGL00000000	current	**+**				**+**	
Ent-IA-2010-env swab-SEEE0816	Enteritidis str. 607308-16	USA:IA	Environmental Swab	2010-08-19	JEGX01.0004_JEGA26.0002	52623	ALGM00000000	current	**+**			**+**	**+**	
Ent-IA-2010-env swab-SEEE0819	Enteritidis str. 607308-19	USA:IA	Environmental Swab	2010-08-19	JEGX01.0004_JEGA26.0002	52625	ALGN00000000	current	**+**			**+**	**+**	
Ent-IA-2010-env swab-SEEE3072	Enteritidis str. 607307-2	USA:IA	Environmental Swab	2010-08-16	JEGX01.0004_JEGA26.0031	52627	ALGO00000000	current	**+**				**+**	**+**
Ent.PT8-AZ-1977-clinical-SEEE0424	Enteritidis str. CDC 77-0424	USA:AZ	Clinical	1977	JEGX01.0004_JEGA26.0002	53259	ALEL00000000	(Timme, 2012)	**+**				**+**	
Ent-IA-2010-env swab-SEEE3089	Enteritidis str. 607308-9	USA:IA	Environmental Swab	2010-08-19	JEGX01.0004_JEGA26.0002	53261	ALGP00000000	current	**+**			**+**	**+**	
Ent-IA-2010-env swab-SEEE3076	Enteritidis str. 607307-6	USA:IA	Environmental Swab	2010-08-16	JEGX01.0004_JEGA26.0031	53263	ALEM00000000	(Timme, 2012)	**+**				**+**	**+**
Ent-USA-2010-env swab-SEEE9163	Enteritidis str. 629163	USA	Environmental Swab	2010-08-24	JEGX01.0004_JEGA26.0031	53265	ALGQ00000000	current	**+**				**+**	**+**
Ent-IA-2010-env swab-SEEE4917	Enteritidis str. 485549-17	USA:IA	Environmental Swab	2010-08-30	JEGX01.0004_JEGA26.0030	59531	ALEN00000000	(Timme, 2012)	**+**				**+**	
Ent-IA-2010-env swab-SEEE6622	Enteritidis str. 596866-22	USA:IA	Environmental Swab	2010-08-31	JEGX01.0004_JEGA26.0002	59533	ALEO00000000	(Timme, 2012)	**+**				**+**	
Ent-IA-2010-env swab-SEEE6670	Enteritidis str. 596866-70	USA:IA	Environmental Swab	2010-08-31	JEGX01.0004_JEGA26.0002	59535	ALEP00000000	(Timme, 2012)	**+**				**+**	
Ent-IA-2010-env swab-SEEE6426	Enteritidis str. 629164-26	USA:IA	Environmental Swab	2010-08-30	JEGX01.0034_JEGA26.0002	59537	ALEQ00000000	(Timme, 2012)	**+**					
Ent-IA-2010-env swab-SEEE6437	Enteritidis str. 629164-37	USA:IA	Environmental Swab	2010-08-30	JEGX01.0004_JEGA26.0030	59539	ALER00000000	(Timme, 2012)	**+**				**+**	
Ent-IA-2010-env swab-SEEE7246	Enteritidis str. 639672-46	USA:IA	Environmental Swab	2010-08-31	JEGX01.0004_JEGA26.0002	59541	ALES00000000	(Timme, 2012)	**+**				**+**	
Ent-IA-2010-env swab-SEEE7250	Enteritidis str. 639672-50	USA:IA	Environmental Swab	2010-08-31	JEGX01.0004_JEGA26.0002	59543	ALET00000000	(Timme, 2012)	**+**				**+**	
Ent.PT8-ME-poultry environment-SEEE151	Enteritidis str. SE15-1	USA:ME	Poultry Environment	-	JEGX01.0004_JEGA26.0002	59987	ALGR00000000	current	**+**				**+**	
Ent-USA-2004-chicken-SEEEN202	Enteritidis str. CVM N202	USA	Chicken	2004	JEGX01.0004_JEGA26.0002	59989	ALGS00000000	current	**+**				**+**	
Ent-USA-1956-clinical-SEEE3991	Enteritidis str. CDC 56-3991	USA:TN	Clinical	1956	JEGX01.0004_JEGA26.0002	59991	ALGT00000000	current	**+**				**+**	
Ent-USA-1976-clinical-SEEE3618	Enteritidis str. CDC 76-3618	USA:AZ	Clinical	1976	JEGX01.0004_JEGA26.0002	59993	ALGU00000000	current	**+**				**+**	
Ent.PT8-IA-chicken breast-SEEE1831	Enteritidis str. 13183-1	USA:IA	Chicken Breast	-	JEGX01.0004_JEGA26.0002	59995	ALGV00000000	current	**+**				**+**	
Ent-USA-1981-SEEE2490	Enteritidis str. CDC 81-2490	USA:NJ	Clinical	1981	JEGX01.0004_JEGA26.0002	59997	ALGW00000000	current	**+**				**+**	
Ent-NC-SEEEL909	Enteritidis str. SL909	USA:NC	-	-	NA_NA	59999	ALGX00000000	current	**+**		**+**		**+**	**+ (partial)**
Ent-NC-SEEEL913	Enteritidis str. SL913	USA:NC	-	-	NA_NA	60001	ALGY00000000	current	**+**				**+**	
Ent.PT8-RI-1977-clinical-SEEE1427	Enteritidis str. CDC 77-1427	USA:RI	Clinical	1977	JEGX01.0004_JEGA26.0002	60069	ALEU00000000	(Timme, 2012)	**+**				**+**	
Ent.PT8-SD-1977-clinical-SEEE2659	Enteritidis str. CDC 77-2659	USA:SD	Clinical	1977	JEGX01.0004_JEGA26.0002	60071	ALEV00000000	(Timme, 2012)	**+**				**+**	
Ent.PT8-NB-1978-clinical-SEEE1757	Enteritidis str. CDC 78-1757	USA:NE	Clinical	1978	JEGX01.0004_JEGA26.0002	60073	ALEW00000000	(Timme, 2012)	**+**				**+**	
Ent.PT8-NC-chicken-SEEE5101	Enteritidis str. 22510-1	USA:NC	Chicken	-	JEGX01.0004_JEGA26.0002	60075	ALEX00000000	(Timme, 2012)	**+**				**+**	
Ent-USA-1969-clinical-SEEE4941	Enteritidis str. CDC 69-4941	USA:NH	Clinical	1969	JEGX01.0004_JEGA26.0002	60509	ALGZ00000000	current	**+**				**+**	
Ent-GA-SEEE8B1	Enteritidis str. 8b-1	USA:GA	-	-	NA_NA	60511	ALEY00000000	(Timme, 2012)	**+**				**+**	
Ent-IA-2010-fresh shelled eggs-SEEE7015	Enteritidis str. 638970-15	USA:IA	Fresh Shelled Eggs	2010-10-18	JEGX01.0004_JEGA26.0002	61365	ALHA00000000	current	**+**			**+**	**+**	
Ent.PT2-IA-2001-clinical-SEEE7927	Enteritidis str. 17927	USA:IA	Clinical	2001-07-26	JEGX01.0002_NA	62793	ALHB00000000	current		+			**+**	
Ent.PT21-China-chicken-SEEECHS4	Enteritidis str. CHS4	China	Chicken	-	NA_NA	62795	ALHC00000000	current		+				
Ent.PT13a-IA-2001-clinical-SEEE2558	Enteritidis str. 22558	USA:IA	Clinical	2001-09-13	JEGX01.0026_NA	62797	ALHD00000000	current	**+**					
Ent-OH-2010-env swab-SEEE2217	Enteritidis str. 543463 22-17	USA:OH	Environmental Swab	2010-09-27	JEGX01.0004_JEGA26.0002	62799	ALHE00000000	current	**+**			**+**	**+**	
Ent-OH-2010-env swab-SEEE4018	Enteritidis str. 543463 40-18	USA:OH	Environmental Swab	2010-09-27	JEGX01.0007_JEGA26.0002	62801	ALHF00000000	current	**+**			**+**	**+**	
Ent-OH-2010-env swab-SEEE6211	Enteritidis str. 561362 1-1	USA:OH	Environmental Swab	2010-09-23	JEGX01.0004_JEGA26.0002	62803	ALHG00000000	current	**+**			**+**	**+**	
Ent-OH-2010-env swab-SEEE4441	Enteritidis str. 642044 4-1	USA:OH	Environmental Swab	2010-09-23	JEGX01.0004_JEGA26.0002	62805	ALHH00000000	current	**+**			**+**	**+**	
Ent-OH-2010-env swab-SEEE4647	Enteritidis str. 642046 4-7	USA:OH	Environmental Swab	2010-10-18	JEGX01.0034_JEGA26.0002	62807	ALHI00000000	current	**+**			**+**		
Ent-OH-2010-env swab-SEEE9845	Enteritidis str. 648898 4-5	USA:OH	Environmental Swab	2010-09-24	JEGX01.0004_JEGA26.0002	62809	ALHJ00000000	current	**+**			**+**	**+**	
Ent-OH-2010-env swab-SEEE9317	Enteritidis str. 648899 3-17	USA:OH	Environmental Swab	2010-09-27	JEGX01.0004_JEGA26.0002	62811	ALHK00000000	current	**+**				**+**	
Ent-OH-2010-env swab-SEEE0116	Enteritidis str. 648900 1-16	USA:OH	Environmental Swab	2010-09-27	JEGX01.0007_JEGA26.0002	62813	ALHL00000000	current	**+**			**+**	**+**	
Ent-OH-2010-env swab-SEEE1117	Enteritidis str. 648901 1-17	USA:OH	Environmental Swab	2010-09-27	JEGX01.0004_JEGA26.0002	62815	ALHM00000000	current	**+**			**+**	**+**	
Ent-OH-2010-env swab-SEEE1392	Enteritidis str. 648901 39-2	USA:OH	Environmental Swab	2010-09-27	JEGX01.0004_JEGA26.0011	62817	ALHN00000000	current	**+**				**+**	
Ent-OH-2010-env swab-SEEE0268	Enteritidis str. 648902 6-8	USA:OH	Environmental Swab	2010-10-05	JEGX01.0004_JEGA26.0149	62819	ALHO00000000	current	**+**			**+**	**+**	
Ent-OH-2010-env swab-SEEE0316	Enteritidis str. 648903 1-6	USA:OH	Environmental Swab	2010-10-05	JEGX01.0004_JEGA26.0002	62821	ALHP00000000	current	**+**			**+**	**+**	
Ent-OH-2010-env swab-SEEE0436	Enteritidis str. 648904 3-6	USA:OH	Environmental Swab	2010-10-05	JEGX01.0004_JEGA26.0002	62823	ALHQ00000000	current	**+**			**+**	**+**	
Ent-OH-2010-env swab-SEEE5518	Enteritidis str. 648905 5-18	USA:OH	Environmental Swab	2010-10-05	JEGX01.0004_JEGA26.0002	62825	ALEZ00000000	(Timme, 2012)	**+**				**+**	
Ent-OH-2010-env swab-SEEE1319	Enteritidis str. 653049 13-19	USA:OH	Environmental Swab	2010-10-19	JEGX01.0004_JEGA26.0002	62827	ALHR00000000	current	**+**			**+**	**+**	
Ent-OH-2010-env swab-SEEE1618	Enteritidis str. 648901 6-18	USA:OH	Environmental Swab	2010-09-27	JEGX01.0004_JEGA26.0002	62829	ALFA00000000	(Timme, 2012)	**+**			**+**	**+**	
Ent-OH-2010-env swab-SEEE4481	Enteritidis str. 642044 8-1	USA:OH	Environmental Swab	2010-09-23	JEGX01.0004_JEGA26.0002	62831	ALHS00000000	current	**+**			**+**	**+**	
Ent-OH-2010-env swab-SEEE6297	Enteritidis str. 561362 9-7	USA:OH	Environmental Swab	2010-09-23	JEGX01.0004_JEGA26.0002	62833	ALHT00000000	current	**+**				**+**	
Ent-OH-2010-env swab-SEEE4220	Enteritidis str. 543463 42-20	USA:OH	Environmental Swab	2010-09-27	JEGX01.0004_JEGA26.0002	62835	ALHU00000000	current	**+**			**+**	**+**	
Ent-OH-2010-env swab-SEEE1616	Enteritidis str. 648901 16-16	USA:OH	Environmental Swab	2010-09-27	JEGX01.0004_JEGA26.0002	62837	ALHV00000000	current	**+**			**+**	**+**	
Ent.PT13a-MD-1976-clinical-SEEE2651	Enteritidis str. CDC 76-2651	USA:MD	Clinical	1976	JEGX01.0004_JEGA26.0030	63673	ALHW00000000	current	**+**				**+**	
Ent.PT13-MD-chicken breast-SEEE3944	Enteritidis str. 33944	USA:MD	Chicken Breast	-	JEGX01.0185_JEGA26.0004	63675	ALHX00000000	current	**+**				**+**	
Pullorum-Brazil-SEEP9120	Pullorum str. ATCC 9120	-	-	-	NA_NA	66671	AMYM00000000	current						
Ent.PT8-PA-SEEE5621	Enteritidis str. 6.0562-1	USA:PA	-	-	JEGX01.0004_JEGA26.0002	66681	ALHZ00000000	current	**+**				**+**	
Ent.PT8-TX-1950-clinical-SEEE5646	Enteritidis str. CDC 50-5646	USA:TX	-	1950	JEGX01.0004_JEGA26.0002	66683	ALIA00000000	current	**+**				**+**	
Ent.PT8-NM-1981-clinical-SEEE2625	Enteritidis str. CDC 81-2625	USA:NM	Clinical	1981	NA_NA	72649	ALIB00000000	current	**+**				**+**	
Ent.PT13a-MA-1962-clinical-SEEE1976	Enteritidis str. CDC 62-1976	USA:MA	Clinical	1962	JEGX01.0004_NA	72651	ALIC00000000	current	**+**				**+**	
Ent.PT8-NJ-1950-clinical-SEEE3079	Enteritidis str. CDC 50-3079	USA:NJ	-	1950	NA_JEGA26.0002	73685	ALFB00000000	(Timme, 2012)	**+**				**+**	
Ent.PT8-CA-1953-turkey-SEEE3407	Enteritidis str. CDC 53-407	USA:CA	Clinical	1953	JEGX01.0004_NA	77693	ALID00000000	current	**+**				**+**	

Details for the mobile elements are as follows: RE-2010 Phage (HM770079); Partial Homology to pOU1114 (DQ115387); ALFR00000000 putative plasmid pSEEE1729_15; ALGD00000000 putative plasmid pSEEE0956_35 (Nearly identical to HE663166 and JN885080); ALGO00000000 putative plasmid pSEEE3072_19 (Similar to CP003417).

### Growth of bacterial strains, and genomic and plasmid DNA isolation

Genomic DNA was isolated from overnight cultures as follows: each initial pure culture sample was taken from frozen stock, plated on Trypticase Soy Agar, and incubated overnight at 37°C. After incubation, cells were taken from the plate and inoculated into Trypticase Soy Broth culture for DNA extraction. All samples were representative cultures from a full-plate inoculation and were not single colonies. Genomic DNA was extracted using Qiagen DNeasy kits.

### Library construction and genome sequencing

For this study, all *S.* Enteritidis isolates were shotgun sequenced using the Roche 454 GS Titanium NGS technology [50]. This platform provided longer read lengths relative to other sequencing methods and has a relatively shorter time to generate raw sequence information. Taxon sampling included one new isolate each of *S.* Gallinarum and *S.* Gallinarum biovar Pullorum, two isolates of *S.* Dublin and 106 new isolates of *S.* Enteritidis including a few isolates differing by PFGE patterns, and the majority of isolates sharing the same PFGE pattern (Table 1). These *Salmonella* serotypes have been considered to be close relatives traditionally. Each isolate was run on a quarter of a titanium plate that produced roughly 250,000 reads per draft genome resulting in an average genome coverage of about 20×.

### Genome assembly and annotation

De novo assemblies were created for each *S.* Enteritidis isolate using the Roche Newbler run Assembly software (v. 2.6). All draft genomes were annotated using NCBI's Prokaryotic Genomes Automatic Annotation Pipeline (PGAAP, [51]). Comparison of the de novo assemblies against the complete genome for *S.* Enteritidis strain 125109 (GenBank accession: AM933172) using Mauve [52] identified several large contigs that did not map to the reference genome: phage RE-2010 (Accession: HM7700079), plasmid pOU1114 (Accession: DQ115387, strain SL909), plasmid from strain CDC_2010K_1729 (pSEEE1729_15), plasmid from strain CDC_2010K-0956 (pSEEE0956_35), and plasmid from strain 607307-2 (pSEEE3072_19). The reference sequence used for mapping reads was comprised of the complete *S.* Enteritidis genome (AM933172, which includes the P125109 phage) plus the 5 additional elements previously described.

### Comparative genomic analysis

SNPs were identified by mapping the 454 reads to the reference genome using Roche Newbler runMapping software (v. 2.6). SNPs were defined as positions where one or more isolates differed from the reference sequence with coverage ≥4× and with ≥95% of the reads containing the SNP, excluding insertions and deletions [indels] The alignments were then screened to find non-gap phylogenetically informative nucleotide positions (*i.e.* minor allele count ≥2). The mapped consensus base for each isolate at the reference SNP positions were then concatenated in a multiple FASTA file for phylogenetic analysis. The maximum likelihood tree was constructed using GARLI [53] with 1000 bootstrap replicates. All GARLI analyses were performed with the default parameter settings and the GTR+Γ+I nucleotide substitution model. SNPs in single copy protein coding genes were identified using the same criteria by mapping the isolate reads to the annotated CDS regions in AM933172. Multiple alignments for genes with SNPs were created using the UCLUST [54] software package. There were 366 genes that met the SNP criteria that were present in 95% or more of the 106 isolates. These 366 genes represent a conservative estimate of the set of variable genes as we have eliminated indels and CDS regions that could not be reliably predicted and annotated. A phylogenetic tree also was built with TNT [55] and characters were optimized onto the tree to assess character evolution for several of the critical nodes on the tree associated with the outbreak implicated farm isolates [56] as well as for identifying SNPs specific to *S.* Enteritidis.

Phylogenetic analyses of the clonal *S.* Enteritidis data set including multiple outgroups were performed on the concatenated informative SNP matrix described above. Approximately 99% of the sites in the 5MB *Salmonella* genomes are phylogenetically uninformative (i.e. showing no differences that provide clustering information) and eliminating them dramatically reduces computation time and memory requirements. Additional, phylogenetic analyses were performed on the set of 366 concatenated genes containing informative SNPs.

### Accessions

Whole genome shotgun accessions (WGS), bioproject accession numbers are listed in Table 1.

## Results

### Genome size, order and conservation

**Figure 1:**
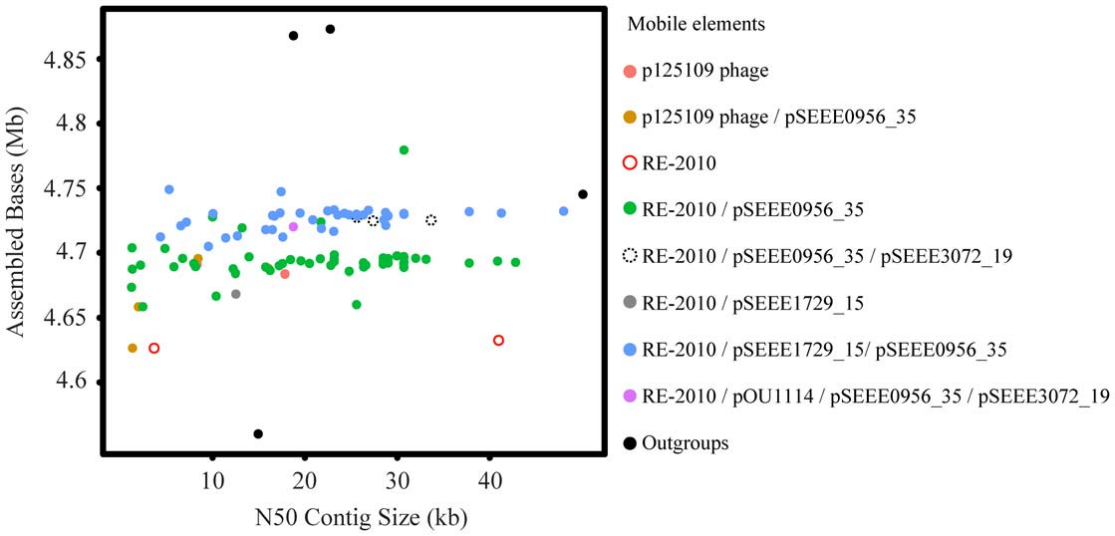
The number of assembled bases and N50 contig size listed for each of the sequenced isolates. Points are colored according to the phages and plasmids that were found in the sequencing results.

**Figure 2:**
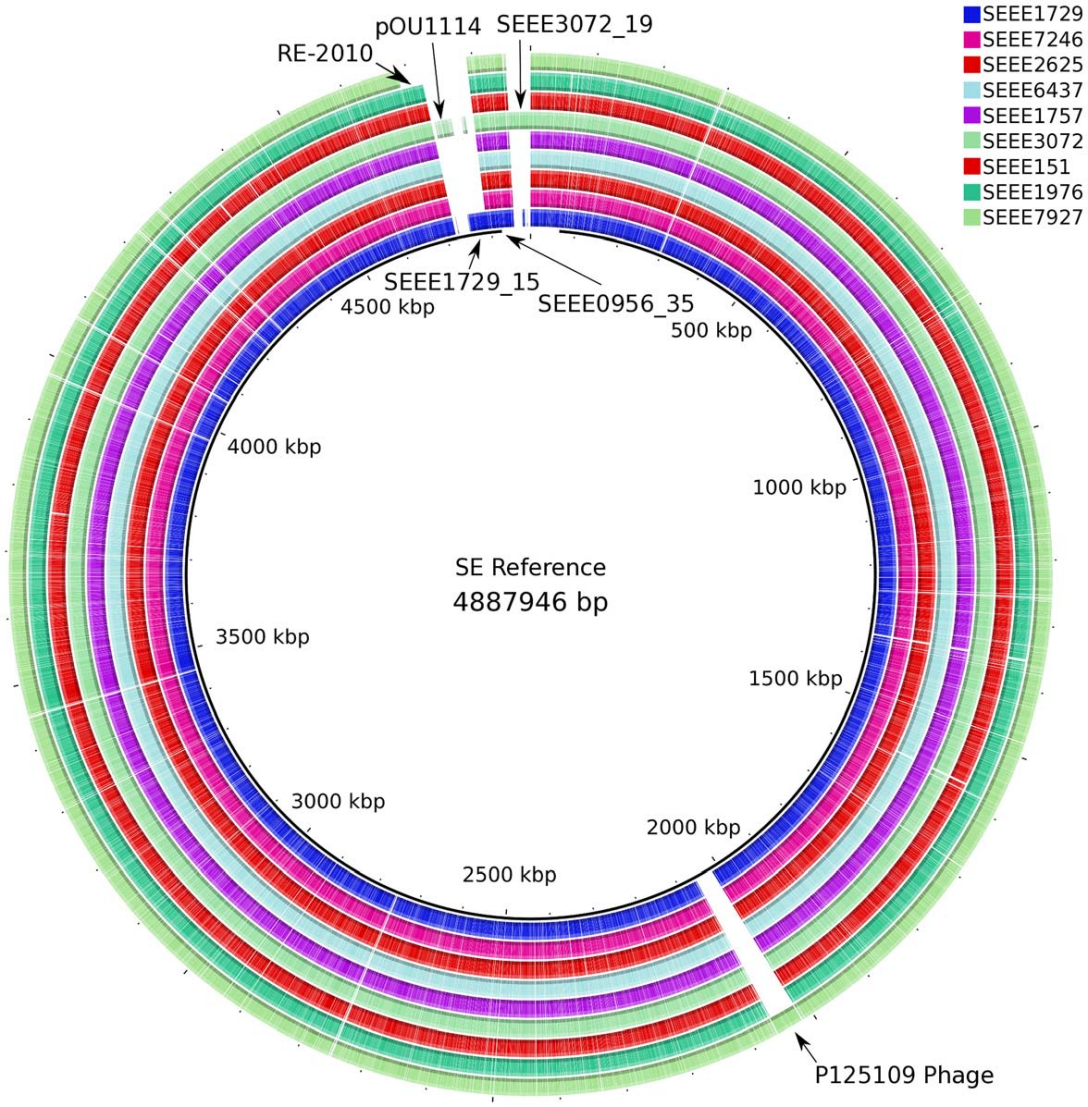
Circle plot showing general conservation of synteny among PFGE pattern JEGX01.0004 of *Salmonella* Enteritidis, with phage and plasmid differences listed for 9 representative isolates.

**Figure 3:**
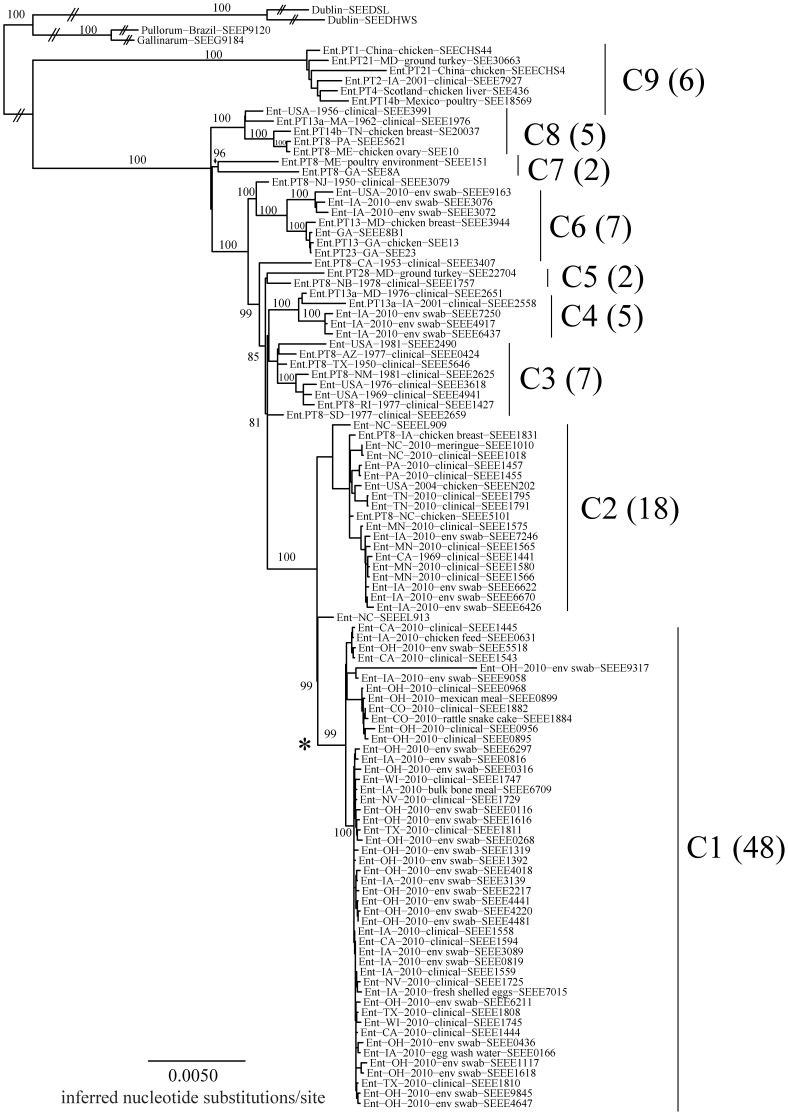
Phylogenetic tree based on the maximum-likelihood method implemented in GARLI. Numbers associated with branches represent the percent of 1000 bootstrap replicates supporting the major clades C1 through C9. Acquisition of ALFR00000000 putative plasmid pSEEE1729_15 is defined by a star at the base of C1.

New draft genomes are provided for 110 *Salmonella* isolates including 106 *S.* Enteritidis, and four closely related outgroups, two *S.* Dublin and one each of *S.* Gallinarum, and *S.* Gallinarum biovar Pullorum (Table 1). While synteny and genome organization were largely stable among these isolates, genome size differences were observed due to variation in the presence or absence of several phages and plasmids including phage RE-2010 [57], phage P125109 [11], plasmid pOU1114 [58], and several newly observed plasmid mobile elements pSEEE1729_15, pSEEE0956_35 and pSEEE3072_19 (Figs. 1 and 2, Table 1). One of these, pOU1114, is a newly finished complete plasmid known from partial data to reside within *S.* Enteritidis and its close relative *S.* Dublin. pSEEE3072_19 is closely related to the previously characterized *S.* Enteritidis plasmid pSENV [59]. Presence or absence of mobile elements in *S.* Enteritidis contributed to a genome size ranging from 4.6 to 4.9 mbp, with *S.* Dublin being relatively larger (∼4.9 mbp) and *S.* Gallinarum smaller (4.55 mbp) when compared to the *S.* Enteritidis genomes collected here. A bimodal split centered on 4.7 mbp was noted, which largely corresponds to mobile elements that partition predictably between phylogenetic lineages (Table 1, Figures 1, 3).

### Most clinical isolates are phylogenetically close to isolates from two egg farms

A set of 106 ecologically diverse food, environmental, and clinical *S.* Enteritidis strain isolates, associated with the time period surrounding the 2010 egg contamination event, were included for whole genome sequencing. Strains with expanding diversity and representing three important levels for comparison were included in the analysis. The first group of 60 strains represented a highly homogeneous set of environmental, farm, food, and clinical *S.* Enteritidis isolates sharing a common PFGE pattern and temporally associated with the 2010 egg contamination event. The second tier of 30 strains included a set of historical environmental, food, and clinical *S.* Enteritidis isolates that retained identical or highly similar PFGE patterns but were unassociated with the 2010 egg contamination event, unrelated in time, location or isolation source. Finally, the last group of 16 isolates was also unrelated to the 2010 egg event and included a series of *S.* Enteritidis strains with more diverged PFGE patterns and phage types away from the 2010 egg *S.* Enteritidis isolates. These strains served largely as genetic references, effectively allowing for a testing of the phylogenetic monophyly of the 2010 egg-associated *S.* Enteritidis isolates. As an example, these isolates include other phage types such as PT4, PT23, PT14b, and PT1 and date back over 50 years in time.

Phylogenetic analysis of these genomes revealed several interesting observations. First, the *S.* Enteritidis PFGE Pattern JEGX01.0004 plus related strains and strains with similar PFGE patterns formed a monophyletic group distinct from other neighboring serovars *S.* Dublin, *S.* Gallinarum, and *S.* Gallinarum biovar Pullorum. Previous comparative genomics studies [12], [14]–[17] have shown that *S.* Enteritidis, *S.* Dublin, *S.* Gallinarum biovar Pullorum and *S.* Gallinarum form a natural group, a finding supported by our results. Second, within *S.* Enteritidis, nine lineages were defined from the tree (Figure 3). Genetic diversity between different serovars included thousands of differences while variability between the nine lineages of *S.* Enteritidis labeled C1–C9, ranged only in the order of 100 to 600 nucleotide changes. Within lineage variation was usually less than 100 bp with the exception of lineage C7 which had over 200 bp of intra-clade variability (Table 2).

**Table 2:** Pairwise SNP distances+/−SD between major lineages identified in the phylogenetic tree (C = clade).

Pairwise SNP distances									
	C1	C2	C3	C4	C5	C6	C7	C8	C9
**C1**	30 (2)								
**C2**	122 (10)	32 (2)							
**C3**	172 (9)	162 (10)	72 (5)						
**C4**	201 (12)	200 (14)	145 (11)	63 (8)					
**C5**	176 (8)	175 (10)	122 (8)	152 (9)	117 (8)				
**C6**	212 (11)	209 (13)	155 (11)	187 (11)	160 (9)	58 (6)			
**C7**	272 (12)	270 (13)	215 (9)	246 (12)	216 (8)	225 (8)	205 (15)		
**C8**	253 (10)	252 (14)	199 (8)	229 (12)	199 (10)	208 (7)	205 (10)	73 (6)	
**C9**	546 (17)	542 (20)	487 (19)	518 (15)	493 (18)	550 (20)	495 (16)	479 (18)	79 (6)

Among the isolates compared, results for clinical isolates sorted into each of the major lineages (Clades C1, C2, C3 and C5, Figure 3) with most falling into clades C1 and C2. It is noteworthy that no apparent increase in substitutions was observed for the isolates that passed through patients compared to their environmental clones. If there was an increase or expansion in genetic diversity among the clinical isolates studied, compared to other food and environmental *S.* Enteritidis collected in relation to the 2010 egg event, one would expect observed genetic diversity to have been expressed as increased or longer branch lengths among the terminal tree nodes leading to the 2010 clinical isolates in the tree. In general, this was not observed. Albeit, several clinical isolates (i.e., SEEE9845 and SEEE4647 both from Ohio) reflect the accumulation of just a few additional SNPs in the tree as their terminal branches project slightly from the base of the 2010 egg isolates in clade 1. However, comparable subtle genetic variations among environmental and egg isolates were also noted as well in the tree indicating that no additional or overt pressure to change was applied *in vivo* for the clinical strains included here among the 2010 egg and environmental isolates. For example, environmental isolates from Ohio (e.g., SEEE1117 and SEEE1618), also in clade 1, vary comparably in their branch lengths to the aforementioned clinical isolates.

Clades C7, C8 and C9 contained a diversity of isolates from unrelated and historical freezer stocks that were not connected to the large shell egg outbreak (Table 1). Additionally, environmental *S.* Enteritidis isolates taken from Farm 1 were found in clades C6 and C1, while environmental *S.* Enteritidis isolates from Farm 2 were observed in Clades C4, C2 and one isolate in C1. It is important to note that in our *S.* Enteritidis strain tree presented here, the phylogenomic data sort in a largely hierarchical fashion. That is, isolates associated with the 2010 *S.* Enteritidis egg event do cluster most closely together with additional SNP diversity providing higher resolution for related strains within the contamination event. Additionally, nearly all of the reference isolates retaining common PFGE patterns but unassociated with the egg event sort adjacent to but outside of the 2010 *S.* Enteritidis egg, clinical, and farm swarm of isolates. Surprisingly, however, several of these genetically similar *S.* Enteritidis reference strains lacking any temporal relatedness to the 2010 egg event do partition with other egg isolates. One *S.* Enteritidis isolate from 2004, for example, formed a sub-clade with two clinical isolates from Tennessee within the larger clade 2 in the genome tree (Figure 3). Also in clade 2, a historical *S.* Enteritidis isolate from California (1441) sorted closely with two *S.* Enteritidis clinical isolates from Minnesota collected from 2010 and during the egg event. The substantial number of SNPS that partition strains within *S.* Enteritidis clades 1 and 2 and examples of phylogenetic homogeneity may point to additional source reservoirs of *S.* Enteritidis contamination during the 2010 egg event.

It is important to note that many *S.* Enteritidis strains with common phage-types are polyphyletic (do not sort into a single group) in the whole-genome sequence tree. *S.* Enteritidis strains designated as PT8, for example, are phylogenetically distributed across clades 1, 2, 3, 5, 6, 7, and 8 suggesting that despite retaining this common phenotypic feature, phage types are phylogenetically distinct and diverged among their genome sequences. This observation is not unexpected [9] given the intrinsic horizontal movement of phage restriction across diverged strains of *S. enterica*.

### Genetic variation defining *S.* Enteritidis

More than 50 genes vary with SNPs that define *S.* Enteritidis separately from the outgroups compared in this study (Table 3). For example, the multicopper oxidase gene, (*cue*O, locus tag SEN0173), represents one gene with numerous genetic signatures unique to *S.* Enteritidis strains. This gene and protein alignment show a dozen SNP differences and three amino acid differences that appear to be present in all *S.* Enteritidis examined. Serovar-defining signature amino acid differences include E to Q (position 132), P to L (position 337), and L to S changes (position 342). Other genes that vary with *S.* Enteritidis specific SNPs and amino acid changes include: the fimbrial usher protein (*bcf*C, locus tag SEN0022); fimbrial structural subunit (*saf*D, locus tag SEN0284); 2-methylcitrate dehydratase (*prp*D, locus tag SEN0353); Trp operon repressor (*trp*R, locus tag SEN4339); tRNA(Ile)-lysidine synthetase gene (*til*S, locus tag SeD_A0258); iron-hydroxamate transporter ATP-binding subunit (*fhu*C, locus tag STM0192); ABC transporter ATP-binding protein (locus tag SEN0716); electron transfer flavoprotein (*fix*A, locus tag SEN0076); and invasion-associated secreted effector protein (*sop*E2, locus tag SEN1182) to name a few (Table 3).

**Table 3:** Variable genes observed that may define the serotype *Salmonella* Enteritidis.

Variable genes observed that may define Salmonella Enteritidis.									
Number	Gene Alignment	Protein Alignment	Nt Pos	NT Change	AA Pos	AA Change	Gene Symbol	Locus Tag	ReBlasted translated proteins from Next Gen Data against NCBI - Feature Matches
1	10_input.aln	10_protein.fas	1995	T→G	665	S→R	bcfC	SEN0022	ref|YP_002242189.1| fimbrial usher protein [Salmonella enterica subsp. enterica serovar Enteritidis str. P125109] Length = 873
2	1014_input.aln	1014_protein.fas	149	C→T	50	P→L	ppiA	SEN3299	ref|YP_002245364.1| peptidyl-prolyl cis-trans isomerase A [Salmonella enterica subsp. enterica serovar Enteritidis str. P125109] Length = 190
3	11_input.aln	11_protein.fas	1078	G→A	360	A→T	mrcA	SEN3319	ref|YP_002245384.1| peptidoglycan synthetase [Salmonella enterica subsp. enterica serovar Enteritidis str. P125109] Length = 858
4	1103_input.aln	1103_protein.fas	263	G→A	88	R→H	SeD_A0525	SeD_A0525	ref|YP_002214434.1| primosomal replication protein N″ [Salmonella enterica subsp. enterica serovar Dublin str. CT_02021853] ref|ZP_09767739.1|
5	1126_input.aln	1126_protein.fas	184	A→G	62	I→V	SEN2582	SEN2582	ref|YP_002244660.1| hypothetical protein SEN2582 [Salmonella enterica subsp. enterica serovar Enteritidis str. P125109] Length = 151
6	1174_input.aln	1174_protein.fas	332	G→A	111	R→H	folK	SEN0188	ref|YP_002242350.1| 2-amino-4-hydroxy-6-hydroxymethyldihydropteridine pyrophosphokinase str. P125109] Length = 159
7	1195_input.aln	1195_protein.fas	22	G→A	8	V→M	safD	SEN0284	ref|YP_002242438.1| fimbrial structural subunit [Salmonella enterica subsp. enterica serovar Enteritidis str. P125109] Length = 156
8	122_input.aln	122_protein.fas	899	C→T	300	A→V	prpD	SEN0353	ref|YP_002242503.1| 2-methylcitrate dehydratase [Salmonella enterica subsp. enterica serovar Enteritidis str. P125109] Length = 483
9	1256_input.aln	1256_protein.fas	263	T→C	88	V→A	ygdK	SEN2829	ref|YP_002244901.1| hypothetical protein SEN2829 [Salmonella enterica subsp. enterica serovar Enteritidis str. P125109] Length = 147
10	1314_input.aln	1314_protein.fas	262	G/T→A	88	N/Y→D	SEN2998	SEN2998	ref|YP_002245065.1| hypothetical protein SEN2998 [Salmonella enterica subsp. enterica serovar Enteritidis str. P125109] Length = 136
11	1335_input.aln	1335_protein.fas	8	G→A	3	Q→R	SEN0986	SEN0986	ref|YP_002243115.1| hypothetical protein SEN0986 [Salmonella enterica subsp. enterica serovar Enteritidis str. P125109] Length = 131
12	1345_input.aln	1345_protein.fas	227	A→-/G	76	E→-/G	sirC	SEN1265	ref|YP_002243370.1| transcriptional regulator [Salmonella enterica subsp. enterica serovar Enteritidis str. P125109] Length = 129
13	1439_input.aln	1439_protein.fas	11	A→G	4	H→R	trpR	SEN4339	ref|YP_002246355.1| Trp operon repressor [Salmonella enterica subsp. enterica serovar Enteritidis str. P125109] Length = 108
14	1450_input.aln	1450_protein.fas	11	T→G	4	I→S	sugE	STY4698	ref|NP_458777.1| quaternary ammonium compound-resistance protein SugE [Salmonella enterica subsp. enterica serovar Typhi str. CT18]
15	1504_input.aln	1504_protein.fas	55	G/-→A	19	A→T/-	SEN0159	SEN0159	ref|YP_002242321.1| hypothetical protein SEN0159 [Salmonella enterica subsp. enterica serovar Enteritidis str. P125109] Length = 94
16	189_input.aln	189_protein.fas	346	C→T	116	H→Y	dinF	SEN4007	>ref|YP_002246043.1| DNA-damage-inducible SOS response protein [Salmonella enterica subsp. enterica serovar Enteritidis str. P125109]
17	208_input.aln	208_protein.fas	110	G→T	37	R→L	tilS	SeD_A0258	ref|YP_002214197.1| tRNA(Ile)-lysidine synthetase [Salmonella enterica subsp. enterica serovar Dublin str. CT_02021853] ref|ZP_09762840.1|
18	245_input.aln	245_protein.fas	164	C→T	55	A→V	argD	SEN3295	ref|YP_002245360.1| bifunctional N-succinyldiaminopimelate-aminotransferase/acetylornithine transaminase protein str. P125109] Length = 405
19	304_input.aln	304_protein.fas	292	G→A	98	D→N	gldA	SEN3354	ref|YP_002245419.1| glycerol dehydrogenase [Salmonella enterica subsp. enterica serovar Enteritidis str. P125109] Length = 369
20	305_input.aln	305_protein.fas	128	C→T/-	43	P→L/-	phnT	SEN0410	ref|YP_002242560.1| 2-aminoethylphosphonate transporter ATP-binding protein str. P125109] Length = 369
21	310_input.aln	310_protein.fas	694	G→A	232	D→N	yhcG	SEN3165	ref|YP_002245231.1| hypothetical protein SEN3165 [Salmonella enterica subsp. enterica serovar Enteritidis str. P125109] Length = 367
22	312_input.aln	312_protein.fas	70	G→A	24	D→N	SPAB_05003	SPAB_05003	ref|YP_001591127.1| hypothetical protein SPAB_05003 [Salmonella enterica subsp. enterica serovar Paratyphi B str. SPB7] Length = 366
23	32_input.aln	32_protein.fas	103	G→A	35	D→N	SEN3501	SEN3501	ref|YP_002245567.1| hypothetical protein SEN3501 [Salmonella enterica subsp. enterica serovar Enteritidis str. P125109] Length = 651
24	340_input.aln	340_protein.fas	416	G→A	139	G→D	SEN4316	SEN4316	ref|YP_002246331.1| hypothetical protein SEN4316 [Salmonella enterica subsp. enterica serovar Enteritidis str. P125109] Length = 354
25	348_input.aln	348_protein.fas	1022	C→T	341	S→F	SPAB_00445	SPAB_00445	ref|YP_001586711.1| phosphoribosylaminoimidazole synthetase [Salmonella enterica subsp. enterica serovar Paratyphi B str. SPB7]
26	356_input.aln	356_protein.fas	493	G→A	165	D→N	galM	SEN0718	ref|YP_002242862.1| aldose 1-epimerase [Salmonella enterica subsp. enterica serovar Enteritidis str. P125109] Length = 346
27	467_input.aln	467_protein.fas	319	A→C	107	I→L	sinR	STM0304	ref|NP_459302.1| transcriptional regulator [Salmonella enterica subsp. enterica serovar Typhimurium str. LT2] ref|ZP_03076991.1|
28	509_input.aln	509_protein.fas	46	T→G	16	F→V	SEN0315	SEN0315	ref|YP_002242465.1| hydrolase or acyltransferase [Salmonella enterica subsp. enterica serovar Enteritidis str. P125109] Length = 304
29	529_input.aln	529_protein.fas	260	G(TG)→A	87	R/C→H	yihU	SEN3811	ref|YP_002245864.1| oxidoreductase [Salmonella enterica subsp. enterica serovar Enteritidis str. P125109] Length = 298
30	532_input.aln	532_protein.fas	478	G→A	160	A→T	SEN1764	SEN1764	ref|YP_002243862.1| oxidoreductase [Salmonella enterica subsp. enterica serovar Enteritidis str. P125109] Length = 282
31	539_input.aln	539_protein.fas	337	G→A	113	A→T	SEN1001	SEN1001	ref|YP_002243131.1| DNA-binding protein [Salmonella enterica subsp. enterica serovar Enteritidis str. P125109] Length = 296
32	571_input.aln	571_protein.fas	251	C→T	84	P→L	SD3246_2037	SD3246_2037	ref|ZP_09764523.1| aminoglycoside resistance protein [Salmonella enterica subsp. enterica serovar Dublin str. SD3246] Length = 289
33	589_input.aln	589_protein.fas	848	G→A	283	G→E	SEN0539	SEN0539	ref|YP_002242687.1| AraC family transcriptional regulator [Salmonella enterica subsp. enterica serovar Enteritidis str. P125109] Length = 284
34	590_input.aln	590_protein.fas	833	C→T	278	A→V	SEN1713	SEN1713	ref|YP_002243813.1| DNA/RNA non-specific endonuclease [Salmonella enterica subsp. enterica serovar Enteritidis str. P125109] Length = 284
35	61_input.aln	61_protein.fas	599	C→T	200	T→I	cysI	SEN2786	ref|YP_002244858.1| sulfite reductase subunit beta [Salmonella enterica subsp. enterica serovar Enteritidis str. P125109] Length = 570
36	653_input.aln	653_protein.fas	185	C→T	62	P→L	fhuC	STM0192	ref|NP_459197.1| iron-hydroxamate transporter ATP-binding subunit [Salmonella enterica subsp. enterica serovar Typhimurium str. LT2]
37	686_input.aln	686_protein.fas	85	C→T	29	R→C	SEN0716	SEN0716	ref|YP_002242860.1| ABC transporter ATP-binding protein [Salmonella enterica subsp. enterica serovar Enteritidis str. P125109] Length = 258
38	695_input.aln	695_protein.fas	691	G→A	231	E–> K	fixA	SEN0076	ref|YP_002242240.1| electron transfer flavoprotein FixA [Salmonella enterica subsp. enterica serovar Enteritidis str. P125109] Length = 256
39	709_input.aln	709_protein.fas	480	G→T	160	E→D	SEN3371	SEN3371	ref|YP_002245437.1| hypothetical protein SEN3371 [Salmonella enterica subsp. enterica serovar Enteritidis str. P125109] Length = 253
40	710_input.aln	710_protein.fas	458	G→A	153	G→E	stbE	SEN0319	ref|YP_002242469.1| fimbrial chaperone protein [Salmonella enterica subsp. enterica serovar Enteritidis str. P125109] Length = 252
41	727_input.aln	727_protein.fas	253	C→T	85	H→Y	SEN0801	SEN0801	ref|YP_002242941.1| electron transfer flavoprotein subunit beta [Salmonella enterica subsp. enterica serovar Enteritidis str. P125109] Length = 249
42	757_input.aln	757_protein.fas	172	G→A	58	D→N	yehV	STM2160	ref|NP_461105.1| transcriptional repressor [Salmonella enterica subsp. enterica serovar Typhimurium str. LT2]
43	767_input.aln	767_protein.fas	455	G→A	152	G→E	sopE2	SEN1182	ref|YP_002243290.1| invasion-associated secreted effector protein (sopE2) str. P125109] Length = 240
44	77_input.aln	77_protein.fas	1025	T→C	342	L→S	cueO	SEN0173	ref|YP_002242335.1| multicopper oxidase [Salmonella enterica subsp. enterica serovar Enteritidis str. P125109] Length = 536
45	77_input.aln	77_protein.fas	1013	C→T	337	P→L	cueO	SEN0173	ref|YP_002242335.1| multicopper oxidase [Salmonella enterica subsp. enterica serovar Enteritidis str. P125109] Length = 536
46	77_input.aln	77_protein.fas	394	G→C	132	E→Q	cueO	SEN0173	ref|YP_002242335.1| multicopper oxidase [Salmonella enterica subsp. enterica serovar Enteritidis str. P125109] Length = 536
47	793_input.aln	793_protein.fas	250	G→A	84	A→T	minC	SEN1223	ref|YP_002243330.1| septum formation inhibitor [Salmonella enterica subsp. enterica serovar Enteritidis str. P125109] Length = 235
48	796_input.aln	796_protein.fas	6	C→A	2	N→K	yggS	SEN2943	ref|YP_002245012.1| hypothetical protein SEN2943 [Salmonella enterica subsp. enterica serovar Enteritidis str. P125109] Length = 234
49	824_input.aln	824_protein.fas	475	G→C	159	V→L	SEN0992	SEN0992	ref|YP_002243123.1| hypothetical protein SEN0992 [Salmonella enterica subsp. enterica serovar Enteritidis str. P125109] Length = 230
50	826_input.aln	826_protein.fas	152	A→G	51	D→G	ygjQ	SEN3064	ref|YP_002245130.1| hypothetical protein SEN3064 [Salmonella enterica subsp. enterica serovar Enteritidis str. P125109] Length = 230
51	864_input.aln	864_protein.fas	126	A→C	42	E→D	ygiB	SEN3030	ref|YP_002245097.1| hypothetical protein SEN3030 [Salmonella enterica subsp. enterica serovar Enteritidis str. P125109] Length = 223
52	865_input.aln	865_protein.fas	40	G→T	14	A→S	nrfC	SEN4049	ref|YP_002246083.1| cytochrome c-type biogenesis protein [Salmonella enterica subsp. enterica serovar Enteritidis str. P125109] Length = 223
53	882_input.aln	882_protein.fas	347	C→A	116	A→E	nfnB	SEN0548	ref|YP_002242695.1| dihydropteridine reductase [Salmonella enterica subsp. enterica serovar Enteritidis str. P125109] Length = 217
54	903_input.aln	903_protein.fas	580	G→A	194	V→I	SEN4080	SEN4080	ref|YP_002246112.1| hypothetical protein SEN4080 [Salmonella enterica subsp. enterica serovar Enteritidis str. P125109] Length = 217
55	970_input.aln	970_protein.fas	76	A→C	26	I→L	leuD	SEN0111	ref|YP_002242275.1| isopropylmalate isomerase small subunit [Salmonella enterica subsp. enterica serovar Enteritidis str. P125109] Length = 201

### Genetic variation defining *S.* Enteritidis outbreak lineages

At least 366 genes varied among *S.* Enteritidis strains comprising the egg-associated foodborne isolates, the farm environmental samples, and temporally-associated clinical samples (Table S1). Of the 366 genes that varied, 21 had nonsynonymous changes that were optimized to one of the branches supporting egg-associated clades C1, C2 or the shared lineage leading to C1 and C2 collectively (Table 4). These variable genes represent micro-evolutionary changes that arose within this highly clonal lineage of *Salmonella* persisting in the food supply and chicken farm environment; thus they may play a role in the subsequent rapid subtyping of isolates in future food contamination events involving *S.* Enteritidis pattern JEGX01.0004.

**Table 4:** Variable genes observed for several critical outbreak clades.

Variable genes for outbreak clades.									
Alignment	Gene	Genome Acc	Locus Tag	Nuc	AA	Nuc Position	AA Position	Clade	Function
522_output.aln	SthB	SEEE1566	SEEEL909_00972	A/G	G/D	95	32	Clade 2	pili assembly chaperone
785_output.aln	YjjP (NCBI homolog)	SEEEL909	SEEEL909_00762	C/T	I/T	311	104	Clade 2	hypothetical protein - link to 99% Agona
110_output.aln	TdcC	SEEE1747	SEEE1747_16476	C/A	M/L	637	213	Clade1/2	threonine/serine transporter TdcC
132_output.aln	SurA	SEEE1747	SEEE1747_19543	G/A	L	1140	380	Clade1/2	peptidyl-prolyl cis-trans isomerase SurA
222_output.aln	MetN 2 (NCBI homolog)	SEEE1747	SEEE1747_08064	G/C	A/G	959	320	Clade1/2	ABC transporter ATP-binding protein
237_output.aln	FtsH	SEEE1747	SEEE1747_21435	A/T	V/D	869	290	Clade1/2	FtsH protease regulator HflC
262_output.aln	hemH	SEEE1747	SEEE1747_07944	C/T	S/L	317	106	Clade1/2	ferrochelatase
284_output.aln	LysR	SEEE1747	SEEE1747_15296	G/A	P	387	129	Clade1/2	DNA-binding transcriptional regulator LysR
294_output.aln	?	SEEE1747	SEEE1747_16871	A/G	K	15	5	Clade1/2	putative Fe-S oxidoreductase
384_output.aln	mutM (NCBI homolog)	SEEE1747	SEEE1747_06264	C/T	M/T	746	249	Clade1/2	formamidopyrimidine-DNA glycosylase
435_output.aln	BioC	SEEE1747	SEEE1747_12216	C/A	G	399	133	Clade1/2	biotin biosynthesis protein BioC
533_output.aln	PhoP	SEEE1747	SEEE1747_04088	G/A	K/E	268	89	Clade1/2	DNA-binding transcriptional regulator PhoP
7_output.aln	FimD (NCBI homolog)	SEEE1747	SEEE1747_20465	C/A	R/S	912	304	Clade1/2	fimbrial usher protein
79_output.aln	prpD	SEEE1747	SEEE1747_07339	C/T	F	801	267	Clade1/2	prpD|2-methylcitrate dehydratase
812_output.aln	fabZ	SEEE1747	SEEE1747_20248	A/C	D/E	102	34	Clade1/2	fabZ|(3R)-hydroxymyristoyl-ACP dehydratase
457_output.aln	prfH	SEEE1747	SEEE1747_07069	A/C	M/L	49	17	Clade1/2	prfH|peptide chain release factor-like protein
Inform90_output.aln		SEEE1747	SEEE1747_20675	A/G	L	636	212	Clade1/2	sodium-dependent inorganic phosphate symporter
Inform482_output.aln		SEEE1747	SEEE1747_01995	C/T	R/C	175	59	Clade1/2	cytochrome B561
114_output.aln		SEEE1117	SEEE1117_18116	G/A	R	276	92	Clade 1	putative hydrolase
13_output.aln	nrdE (NCBI homolog)	SEEE1117	SEEE1117_03671	C/T	V/A	1451	484	Clade 1	ribonucleoside-diphosphate reductase 2 alpha chain
14_output.aln		SEEE1117	SEEE1117_20925	T/G	R/L	653	218	Clade 1	putative lipoprotein
289_output.aln	prmA	SEEE1117	SEEE1117_19441	A/G	G/D	194	65	Clade 1	ribosomal protein L11 methyltransferase
477_output.aln		SEEE1117	SEEE1117_10493	T/C	A/V	683	228	Clade 1	phosphoribosylaminoimidazole-succinocarboxamidesynthase
505_output.aln	bioD	SEEE1117	SEEE1117_10787	G/A	D/G	128	43	Clade 1	dithiobiotin synthetase
564_output.aln	?	SEEE1117	SEEE1117_12757	G/A	S/G	340	114	Clade 1	hydrolase, carbon-nitrogen family protein
Inform235_output.aln	SEEE1117	SEEE1117	SEEE1117_06937	C/A	A/D	224	75	Clade 1	rare lipoprotein A provisional
678_output.aln		SEEE1117	SEEE1117_08666	C/T	A/V	255	84	Clade 1	putative colanic acid biosynthesis acetyltransferase WcaF

Table includes gene names, locus ID, nucleotide and amino acid changes when they occur, as well as a functional description and notes regarding the changes observed.

### Specific genes associated with implicated farm isolates

Nucleotide substitutions in 17 genes, 11 of which were nonsynonymous were identified at the node uniting isolates from the two egg farms (Table 4). In addition, isolates obtained from Farm 1 shared nonsynonymous changes in two genes *Sth*B and *Yjj*P. Farm 2 *S.* Enteritidis isolates shared substitutions in nine genes, eight of which were nonsynonymous.

## Discussion

Like other molecular epidemiology studies of *Salmonella* employing genomic technologies [19]–[23], this work demonstrates that comparative NGS methods can be employed to clearly augment food contamination investigations by genetically linking the implicated sources of contamination with farm and clinical isolates. The genomic evidence herein corroborates epidemiological conclusions from outbreak investigations based on statistical analysis and source tracking leads. However, with NGS, one can gain additional detailed micro-evolutionary knowledge within the associated outbreak and reference isolates; thus providing additional evidence linking implicated farms to some of the clinical isolates but not others initially associated with this foodborne contamination. Moreover, the level of genetic resolution obtained using NGS methods permits a delimiting of the scope of an outbreak in the context of an investigation even for the most genetically homogeneous salmonellae (e.g., *S.* Enteritidis). In this study, NGS data retrospectively supported the decision to recall a half a billion shell eggs by revealing numerous nucleotide and amino acid changes (SNPs) found in both eggs and from hen houses; the changes were also shared with some food and clinical isolates. It is noteworthy that the comparative NGS results reported here provided additional resolution, with new genomic data, that some clinical isolates collected during the time of the egg contamination event and with the same PFGE Pattern JEGX01.0004 may not be linked to the implicated farm isolates studied. That is, while most of the strains collected during this time period and sharing a common PFGE pattern fall into clades 1 and 2 (Figure 3) with the egg and farm isolates, several strains known to be unrelated to the outbreak, including historical isolates from 2004, interrupt these lineages, indicating additional potential sources of contamination.

Data mining associated with these novel genomes should provide new genetic targets for tool development in public health laboratories and that will augment investigations during highly clonal outbreaks of *Salmonella* pathogens. Akin to earlier findings of NGS-based differentiation of S. Montevideo isolates associated with pepper and spiced meats [19]–[21], the signature genetic differences uncovered here will provide additional insight into what will likely remain a common pattern of *S.* Enteritidis associated with the food supply. This bolus of unique genetic identifiers yielded from whole-genome sequencing clearly earmark NGS as a valuable tool for augmenting future molecular epidemiology investigations both for rapidly distinguishing distinct serotypes and PFGE types as well as providing markers that can differentiate highly clonal outbreak lineages into insightful isolate sublineages.

By using a targeted comparative genomic approach that spanned nearly the entire genomic complement of the highly homogeneous *S.* Enteritidis variants included here (i.e., PFGE pattern JEGX01.0004), a robust genotyping SNP panel was compiled that not only discriminated this S. Enteritidis clone from other closely related strains but also fully resolved member isolates within this cluster. This is an important alternative to other methods that have been examined for surveying genomic diversity among foodborne pathogenic strains. One such approach uses NGS to examine diversity among a pooled isolate set instead of on pure cultures, but as expected, this approach is far less robust. As an example, a recent genotyping panel for 0157 STECs revealed lower diversity among the isolates using the selected NGS-based genotyping panel than a two-enzyme PFGE method [60]. Specifically, the authors reported finding over 16,000 variable SNPs, but by pooling STEC isolates and sequencing at low coverage, critical SNPs defining major lineages and sublineages went undetected in this analysis. This was likely due to the failure of the “pooling” approach to link signature SNPs back to a particular source genome. While strain “pooling” may be a faster way to collect SNP data, it may not be an optimal method when discriminating a specific lineage of strains or an isolate cluster of interest. In contrast, comparative genomics approaches rely on high-coverage draft genomes coupled with rigorous phylogenetic analyses and character optimization to resolve accurate evolutionary and genetic relatedness among closely related strains. With such information, individual SNPs can be evaluated in an evolutionary context (i.e., whether they define lineages or represent homoplasy due to convergent gains or character reversals). Indeed, a targeted phylogenetic approach produces a robust genotyping panel because the resultant SNPs can be carefully chosen to represent diversity among targeted isolates while omitting uninformative SNPs [19]–[21]. Conversely, “pooling” strategies might work better within clonal outbreak lineages where hundreds not thousands of SNPs are present.

Mobile elements, such as phages and plasmids, are often the most promiscuous portions of the bacterial genome including *Salmonella* [61]. The mobilome, as it is often collectively referred, appears to be regularly rearranging among closely related clonal lineages of *Salmonella* [19], [21]. As expected, *S.* Enteritidis shows a similar susceptibility to loss and gain of these elements [62], as do other members of the *Enterobactericeae.* In addition to seeing variability among these elements, several new plasmids were discovered, suggesting that additional mobile elements were previously undescribed across the Salmonella genome. Recent examples of new phages and plasmids are being published regularly [63]–[64]. It is becoming apparent that a renewed effort to describe and identify the complete mobilomes of newly sequenced isolates should be undertaken, especially for pathogenic strains that persist and emanate from the environment. From these data, it would appear that mobility of these elements is not restricted to close members. At least one of the newly discovered *Salmonella* plasmids (pSEEE1729_15) had its closest BLAST match to an *E. coli* 0157:H7 strain EC4115 [26], suggesting that parts of the mobilome may be transferred from other related enterobacterial species. Moreover, observations of this nature clearly broaden the possibility of new acquisitions into the *S.* Enteritidis pan genome [62].

Natural selection has been reported in other *Salmonella* isolates and appears to be a major component of the evolution of this pathogen [18], [22]. Some of the genes that vary are found on the mobilome, such as the putative phage terminase gene, supporting the notion that there are actively evolving genes on some mobile elements. This strategy for evolution could provide a scenario whereby highly selected genes could be shaped by natural selection and then easily distributed among the various members of a serotype and other more distant lineages through mobile genetic elements.

Some investigators are beginning to search for genetic determinants for survival and virulence of *S.* Enteritidis in chickens, mice, and cell culture models. Through observing which genes varied in environmental farm and clinical isolates, such insight was sought in the hopes of identifying potential contributing factors to outbreaks. One study linked SNP variability in a stress response gene (*rpo*S) to isolates able to infect poultry [8]. We observed nonsynonymous variability in a gene (*pho*P) that has been demonstrated to be a regulator of *rpo*S [65], [66] and that gene varied uniquely in the lineage defining Clades 1 and 2 (Table 4). The *pho*P gene also is thought to be important to *S.* Enteritidis virulence based on evidence from a mouse model [67]. This change was observed in the SNPs listed in Table 4, which are a conservative subset of variable SNPs and genes, although these SNPs were chosen for potential diagnostic utility and not for a full description of comparative genomics purposes within these isolates.

Another recent hypothesis for the genes involved in salmonellosis, focuses on the ABC transporter genes and the ability of pathogens to acquire nutrients for survival during host infection [68], [69]. Our study shows variability in an ABC transporter for methionine specific for clades 1 and 2 (Table 4). The *S.* Enteritidis model that Osborne et al. [68] tested for *in vivo* with an ABC transporter of alanine is similar to the natural variability for a similar gene in the implicated farm and associated clinical isolates. If this model, affirmed in cell culture studies, holds in chickens, then infections in chickens and eggs in 2010 may be related to the ability of *S.* Enteritidis to survive in a poultry host due to the enhanced access to methionine. The ABC transporters have been hypothesized to be an important new acquisition for all of subspecies I *Salmonella* enterica [15]. Perhaps the ABC transporter gene gave *Salmonella* subspecies I an overall enhanced ability to survive in a warm blooded vertebrate host, and later mutations of the gene allow some serotypes to have special affinity for one host over another. It is common to see serotype specific *Salmonella* that are more common to one host, such as *S.* Kentucky in cattle and *S.* Enteritidis in poultry and eggs. Another nonsynonymous gene change observed is in the threonine/serine transporter *tdc*C gene (Table 4), demonstrating that several transporter genes are evolving within these critical isolates.

*Salmonella*'s ability to gain access to another valuable resource such as metals, like Fe, Mn, and Zn, may help give this foodborne pathogen a competitive edge in the vertebrate gut [70]. Variability in genes related to metal acquisition may help *Salmonella* bypass a process called nutritional immunity. We see another nonsynonymous change unique to the outbreak-associated isolates in a ferrochelatase gene (*hem*H), lending support to this hypothesis. Another hypothesis, argues that diversification within the *Salmonella* fimbriae gene clusters has been implicated as a source for virulence [71] through possible host specific intestinal adhesion mechanisms. At least three genes from gene complexes (*bcf*C, *saf*D, and *stb*E) show unique amino acid changes that may define *S.* Enteritidis (Table 3) and one fibrial gene (*fim*D) shows a unique amino acid change leading to clades 1 and 2 (Table 4).

The nonsynonymous changes that we see among genes that vary for clades 1 and 2 suggest that there may not be a single cause for increased risk of infection and outbreak stemming from chickens and shell eggs. Rather a combination of several of these genetic factors that raise the risks for *Salmonella* invasion may be causing contaminations in the food supply today. The fact that 5 of the 21 nonsynonymous changes varying among the outbreak isolates (Table 3) are putatively involved in virulence-based pathways strongly suggest that there may be multiple and potentially synergistic causes to the expanding rate of *S.* Enteritidis populations. This also suggests that the other genes (Table 3 and 4) that vary in *S.* Enteritidis should be carefully examined and experimentally tested, as more of these are likely to be associated with an increase in virulence and infection [67], [69], [71].

Based on both PCR and sequencing evidence, numerous studies have found little genetic variation within *S.* Enteritidis [6]–[9]. Our genomic diversity estimates for the *S.* Enteritidis PFGE Pattern JEGX01.0004 examined in this study are consistent with other diversity comparisons described between two *S.* Enteritidis isolates of phage type 13 [7]. This variation was observed both as SNP variation among 366 genes as well as the presence and absence of numerous phages and plasmids among these close relatives. This genetic variability was used to define the most variable genes and to assess population and phylogenetic evolutionary patterns for these important foodborne pathogens. In this study, our comparative genomics approach allowed us to cluster clinical isolates within the context of their environmental source, farm isolates, many of which were associated with a large national shell egg recall. Numerous genetic changes clearly link some clinical and environmental isolates to the farms that were implicated in the recall of over a half a billion eggs. One known plasmid in *S.* Enteritidis was completely sequenced, and three plasmids were reported. Several of the genes that varied with nonsynonymous changes had previously been associated with virulence pathways in prior *in vitro* experiments.

### Availability of data and cultures

All NCBI *S.* Enteritidis isolates are linked to Bioproject and new accession numbers AHUJ00000000- AHUR00000000, ALEA00000000- ALEZ00000000, ALFA00000000- ALFZ00000000, ALGA00000000-ALGZ00000000, ALHA00000000- ALHZ00000000. and ALIA00000000- ALID00000000. Cultures included in this study are also available upon request. Please direct any queries to our strain curator Dwayne Roberson, at Dwayne.Roberson@fda.hhs.gov.

## Supporting Information

Table S1 **Variable genes observed within our sample of** ***Salmonella*** **Enteritidis.**
(DOCX)
