# Supplementary material for: On the Evolutionary History, Population Genetics and Diversity among Isolates of Salmonella Enteritidis PFGE Pattern JEGX01.0004
Source: PLoS One. 2013 Jan 30;8(1):e55254. doi: 10.1371/journal.pone.0055254 (PMC3559427; doi:10.1371/journal.pone.0055254)
Supplement: Table S1 — Variable genes observed within our sample of Salmonella Enteritidis. (DOCX) [file pone.0055254.s001.docx]

Supplemental Table S1. Variable genes observed within our sample of *Salmonella* Enteritidis.
